# Differential Diagnosis between Low-Grade and High-Grade Astrocytoma Using System A Amino Acid Transport PET Imaging with C-11-MeAIB: A Comparison Study with C-11-Methionine PET Imaging

**DOI:** 10.1155/2018/1292746

**Published:** 2018-06-20

**Authors:** Ryuichi Nishii, Tatsuya Higashi, Shinya Kagawa, Maya Arimoto, Yoshihiko Kishibe, Masaaki Takahashi, Shigeki Yamada, Masaaki Saiki, Yoshiki Arakawa, Hiroshi Yamauchi, Chio Okuyama, Masato Hojo, Toshihiro Munemitsu, Masahiro Sawada, Masato Kobayashi, Keiichi Kawai, Shigeki Nagamachi, Toshinori Hirai, Susumu Miyamoto

**Affiliations:** ^1^Department of Molecular Imaging and Theranostics, National Institute of Radiological Sciences (NIRS), QST, 4-9-1 Anagawa, Inage-ku, Chiba 263-8555, Japan; ^2^Department of Radiology, Faculty of Medicine, University of Miyazaki, 5200 Kihara Kiyotake-cho, Miyazaki 889-1692, Japan; ^3^Division of PET Imaging, Shiga Medical Center Research Institute, 5-4-30 Moriyama, Moriyama, Shiga 524-8524, Japan; ^4^Department of Diagnostic Imaging and Nuclear Medicine, Graduate School of Medicine, Kyoto University, 54 Shogoin Kawahara-cho, Sakyo-ku, Kyoto 606-8507, Japan; ^5^Department of Neurosurgery, Rakuwakai Otowa Hospital, 2 Otowachinjicho, Yamashina-ku, Kyoto 607-8062, Japan; ^6^Department of Radiology, Japanese Red Cross Otsu Hospital, 1-1-35 Nagara, Otsu, Shiga 520-8511, Japan; ^7^Department of Neurosurgery, Graduate School of Medicine, Kyoto University, 54 ShogoinKawahara-cho, Sakyo-ku, Kyoto 606-8507, Japan; ^8^Department of Neurosurgery, Shiga Medical Center, 5-4-30 Moriyama, Moriyama, Shiga 524-8524, Japan; ^9^Wellness Promotion Science Center, Institute of Medical, Kanazawa University, 5-10-80 Kodatsuno, Kanazawa, Ishikawa 920-0942, Japan; ^10^Graduate School of Health Sciences, College of Medical, Pharmaceutical and Health Sciences, Kanazawa University, 5-10-80 Kodatsuno, Kanazawa, Ishikawa 920-0942, Japan; ^11^Department of Radiology, Faculty of Medicine, Fukuoka University, 7-45-1 Nanakuma, Jonan-ku, Fukuoka 814-0180, Japan

## Abstract

**Introductions:**

[*N*-methyl-C-11]*α*-Methylaminoisobutyric acid (MeAIB) is an artificial amino acid radiotracer used for PET study, which is metabolically stable in vivo. In addition, MeAIB is transported by system A neutral amino acid transport, which is observed ubiquitously in all types of mammalian cells. It has already been shown that MeAIB-PET is useful for malignant lymphoma, head and neck cancers, and lung tumors. However, there have been no reports evaluating the usefulness of MeAIB-PET in the diagnosis of brain tumors. The purpose of this study is to investigate the efficacy of system A amino acid transport PET imaging, MeAIB-PET, in clinical brain tumor diagnosis compared to [S-methyl-C-11]-L-methionine (MET)-PET.

**Methods:**

Thirty-one consecutive patients (male: 16, female: 15), who were suspected of having brain tumors, received both MeAIB-PET and MET-PET within a 2-week interval. All patients were classified into two groups: Group A as a benign group, which included patients who were diagnosed as low-grade astrocytoma, grade II or less, or other low-grade astrocytoma (*n*=12) and Group B as a malignant group, which included patients who were diagnosed as anaplastic astrocytoma, glioblastoma multiforme (GBM), or recurrent GBM despite prior surgery or chemoradiotherapy (*n*=19). PET imaging was performed 20 min after the IV injection of MeAIB and MET, respectively. Semiquantitative analyses of MeAIB and MET uptake using SUVmax and tumor-to-contralateral normal brain tissue (*T*/*N*) ratio were evaluated to compare these PET images. ROC analyses for the diagnostic accuracy of MeAIB-PET and MET-PET were also calculated.

**Results:**

In MeAIB-PET imaging, the SUVmax was 1.20 ± 1.29 for the benign group and 2.94 ± 1.22 for the malignant group (*p* < 0.005), and the *T*/*N* ratio was 3.77 ± 2.39 for the benign group and 16.83 ± 2.39 for the malignant group (*p* < 0.001). In MET-PET, the SUVmax was 3.01 ± 0.94 for the benign group and 4.72 ± 1.61 for the malignant group (*p* < 0.005), and the *T*/*N* ratio was 2.64 ± 1.40 for the benign group and 3.21 ± 1.14 for the malignant group (*n.s.*). For the analysis using the *T*/*N* ratio, there was a significant difference between the benign and malignant groups with MeAIB-PET with *p* < 0.001. The result of ROC analysis using the *T*/*N* ratio indicated a better diagnosis accuracy for MeAIB-PET for brain tumors than MET-PET (*p* < 0.01).

**Conclusions:**

MeAIB, a system A amino acid transport-specific radiolabeled agents, could provide better assessments for detecting malignant type brain tumors. In a differential diagnosis between low-grade and high-grade astrocytoma, MeAIB-PET is a useful diagnostic imaging tool, especially in evaluations using the *T*/*N* ratio.

**Clinical trial registration:**

This trial was registered with UMIN000032498.

## 1. Introduction

Positron emission tomography (PET) imaging with amino acid analogs has been focused greatly on clinical applications, as it targets increased amino acid transport by tumors [1, 2]. Especially for detecting brain tumors, PET studies with amino acid analogs have been developed [3, 4] to overcome the drawbacks of F-18 FDG (FDG) PET, such as physiological uptake by the brain [5, 6].

As methionine, an essential sulfur amino acid, is necessary for the growth and development of cells, radiolabelled [S-methyl-C-11]-L-methionine (MET), mainly transported by system L amino acid transporters [7, 8], has been clinically used as a tumor-seeking agent for PET imaging for several decades [9]. MET-PET images can visualize not only the population and activity of amino acid transport but also metabolic events inside the body, such as active cell membrane transport, cellular protein synthesis, polyamine synthesis, and *trans*-methylation reactions [10, 11]. However, MET-PET is known to have several drawbacks when diagnosing tumors. MET is unstable in vivo due to the aminotransfer reaction [10] and is excreted into the bile and intestines. In addition, MET-PET shows faint physiological uptake in the brain, strong physiological uptake in the liver and bone marrow, and uptake in certain types of inflammatory changes [12, 13].

[*N*-methyl-C-11]*α*-Methylaminoisobutyric acid (MeAIB) is an artificial amino acid radiotracer used for PET study, which is metabolically stable in vivo [14]. Although MET is transported mainly by system L neutral amino acid transport, MeAIB is transported by system A neutral amino acid transport, which is observed ubiquitously in all types of mammalian cells [11, 15]. It has already been shown that MeAIB is useful for amino acid uptake measurements in skeletal muscle and for the diagnosis of malignant lymphoma and head and neck cancers [14, 16, 17]. We have also been investigating system A amino acid PET molecular imaging with MeAIB to detect tumors and have reported its usefulness in the differential diagnosis of pulmonary and mediastinal mass lesions [18] and prostate cancer [19] in clinical practice.

However, there have been no reports evaluating the usefulness of MeAIB-PET in the diagnosis of brain tumors.

The purpose of this study is to investigate the efficacy of system A amino acid transport PET imaging, MeAIB-PET, in clinical brain tumor diagnosis compared to MET-PET.

## 2. Materials and Methods

### 2.1. Patient Characteristics

From March 2009 to December 2011, 31 consecutive patients (male: 16, female: 15), who were suspected of having brain tumors, received both MeAIB-PET and MET-PET within a 2-week interval. Patients' ages ranged from 5 to 71 years with a mean age of 44.2 ± 18.5, as shown in Table 1. Inclusion criteria for the study were as follows: (1) patients were suspected of having an intraaxial brain tumor (newly detected or recurrent lesions 6 months or more after successful treatment) by CT and MRI (both were performed as routine clinical studies), (2) each patient gave written informed consent and received MeAIB-PET and MET-PET, and (3) results were confirmed pathologically, or by clinical follow-up more than 6 months after the PET studies. Exclusion criteria were as follows: (1) patients with extra-axial tumors such as tumors of the meninges, pituitary tumors, pineal parenchymal tumors, or cranial nerve schwannomas, (2) patients with metastatic brain tumors or lymphoma, and (3) patients who refused to receive MeAIB-PET or MET-PET. Of the 52 patients who received MeAIB-PET with suspected brain tumors from March 2009 to December 2011, 31 patients were included in the present study, while the others were excluded because of the exclusion criteria. According to final diagnosis after surgery or biopsy, all patients who met the criteria were classified into the following two groups: Group A (benign), which included patients who were diagnosed as low-grade astrocytoma, grade II including a case of recurrent grade II glioma or less, or other low-grade astrocytomas (*n*=12; ranging from 5 to 46 years, mean age 32.2 ± 10.0 years; seven males and five females); Group B (malignant), which included patients who were diagnosed as anaplastic astrocytoma, glioblastoma multiforme (GBM), or recurrent GBM despite prior surgery or chemoradiotherapy (*n*=19; ranging from 14 to 71 years, mean age 56.7 ± 16.8 years; nine males and ten females).

This prospective clinical study was approved by our institutional review boards, the Human Study Committee (approval number: #36-04, March 25, 2009) and by the Committee for the Clinical Use of Short-Half Life Radioactive Materials (approval number: #2008-01, November 28, 2008). All enrolled patients or their parents if the patient was under 20 years old received explanations, and then they provided written informed consent regarding this study.

### 2.2. Radiotracers

Production of MeAIB followed a previously described procedure [18]. The radiosynthesis method was based on that proposed by Nagren et al. [20]. Chemicals and solvents were of analytical grade and purchased commercially. [^11^C]MeOTf was bubbled into the reactor of an automated remotely controlled synthesizer module C-11-BII (SHI, Tokyo, Japan) filled with 1 mg of methyl *α*-aminoisobutyrate hydrochloride (6.5 mmol) dissolved in 0.4 ml of methanol/acetone (1/1, v/v) and 3.2 *μ*l of 2,2,6,6,*N*-pentamethyl-piperidine (PMP) at −20°C. Then, the reactor was heated to 80°C for 1 min. After cooling to 25°C, 400 *μ*l of 2 M NaOH was then loaded into the reactor. After heating the mixture for 3 min at 60°C, the hydrolyzed product was diluted with 0.5 ml of HPLC eluent and subsequently transferred to a preparative radio HPLC system consisting of a preparative HPLC pump (PU-980, JASCO), an automated flow-detector-controlled injection system with a 2 ml injection loop, a semipreparative HPLC column (hydrophilic interaction chromatography: HILIC column, Nacalai Tesque, 250 × 10 mm^2^, 5 *μ*m; mobile phase: MeCN/10 mM CH_3_COONH_4_, 4/1, v/v; flow: 8 ml/min), a UV detector (254 nm), and an NaI(Tl) radioactivity detector. The product-containing fraction was then diluted with 10 ml of isotonic saline. The radiochemical purity of the MeAIB was more than 99%.

MET was synthesized based on the method described in a previous report [21], by the reaction of C-11 methyltriflate with an aqueous solution of L-homocysteine thiolactone in a Sep-Pak tC18 cartridge, followed by purification with ion-exchange cartridges. The radiochemical purity of MET was also more than 99%.

### 2.3. PET Study

All patients were examined with a whole-body PET scanner, GE Advance (GE Healthcare, Waukesha, WI, USA), or with a whole-body PET/CT scanner, Siemens True Point Biograph 16 (Siemens/CTI, Erlangen, Germany). All subjects received an intravenous injection of MeAIB (513.6 ± 65.6 MBq) or MET (533.9 ± 35.0 MBq). Brain PET/CT images were acquired 20 min after the radiotracer injection in 1 bed position in both study. Emission images were acquired for 5 min per bed position. The data were reconstructed using the ordered subsets expectation-maximization method using eight subsets, two iterations, and an array size of 256 × 256. For the attenuation correction of PET/CT fusion images, the CT component was performed according to a standard protocol with the following parameters: 140 kV; 50 mAs; tube rotation time, 0.5 s per rotation; slice thickness, 5 mm; and gap, 2 mm. An E-soft workstation (Siemens, Nashville, TN, USA) was used to construct PET/CT fusion images.

### 2.4. Image Analysis

PET images were interpreted and analyzed by two experienced nuclear medicine physicians with all the available clinical information, and then a final diagnosis was made in agreement. All PET images were fused with the MRI of each subject using the PMOD software, version 3.1 (PMOD; Zürich, Switzerland). We manually placed an irregular region of interest (ROI) on the coregistered MRI image of each patient, and then these ROIs were transferred to the PET image for the interpretation and calculation of the uptake of each radiotracer. The maximum standardized uptake value (SUVmax) was calculated for semiquantitative analysis of MeAIB and MET uptake by the lesion. The tumor-to-contralateral normal brain tissue (*T*/*N*) ratio was determined by dividing the tumor SUVmax by the SUVmean of the contralateral hemisphere.

### 2.5. Statistics

All values are expressed as mean ± SD. All the statistical analyses were performed using statistical software, JMP version 12 (SAS Institute, Cary, NC, USA), in which *p* values < 0.05 were considered to be statistically significant. A comparison between each group was analyzed with the Wilcoxon score for the unpaired data.

## 3. Results

### 3.1. Characteristics of Patients and Lesions

Final diagnosis was confirmed pathologically by surgical resection, stereotactic biopsy, or by follow-ups of at least more than 6 months. In the benign group of 12 patients, there were 11 astrocytoma grade II or less and one brain stem glioma. In the malignant group of 19 patients, there were 7 with newly diagnosed GBM, 10 with recurrent GBM, and 2 with anaplastic astrocytoma (Table 1).

### 3.2. Visual and Semiquantitative Analysis of MeAIB and MET Uptake

Table 2 summarizes the SUVmax and *T*/*N* ratio of MeAIB- and MET-PET in all patients. In MeAIB-PET imaging, the average SUVmax was 1.20 ± 1.29 for the benign group and 2.94 ± 1.22 for the malignant group (*p* < 0.005), and the average *T*/*N* ratio was 3.77 ± 2.39 for the benign group and 16.83 ± 2.39 for the malignant group (*p* < 0.001). In MET-PET, the average SUVmax was 3.01 ± 0.94 for the benign group and 4.72 ± 1.61 for the malignant group (*p* < 0.005), and the average *T*/*N* ratio was 2.64 ± 1.40 for the benign group and 3.21 ± 1.14 for the malignant group (*n.s.*).

**Figure 1:**
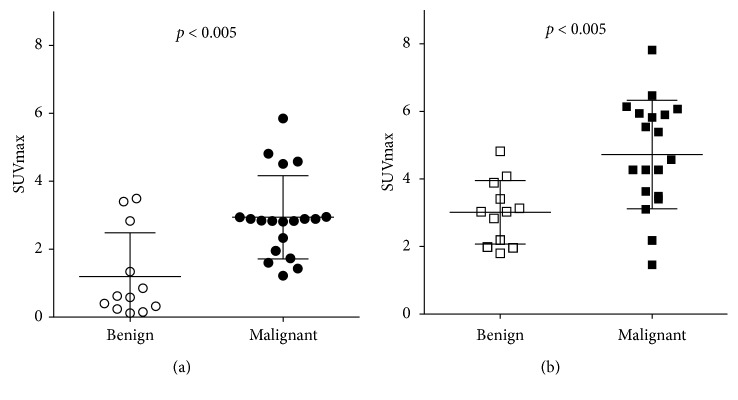
Comparison between benign and malignant groups by SUVmax of the lesions in MeAIB-PET (a) and MET-PET (b).

The average SUVmax of tumors with MeAIB-PET was significantly lower than that with MET-PET. However, MeAIB uptake in the tumors by the malignant group and the benign group showed significant statistical differences with *p* < 0.005 (Figure 1(a)). The average SUVmax of MET in the tumors of the malignant group was significantly higher than that of the benign group *p* < 0.005; however, there was a wide overlap in MET uptake between the benign and malignant groups, resulting in many false positive cases with MET-PET (Figure 1(b)).

**Figure 2:**
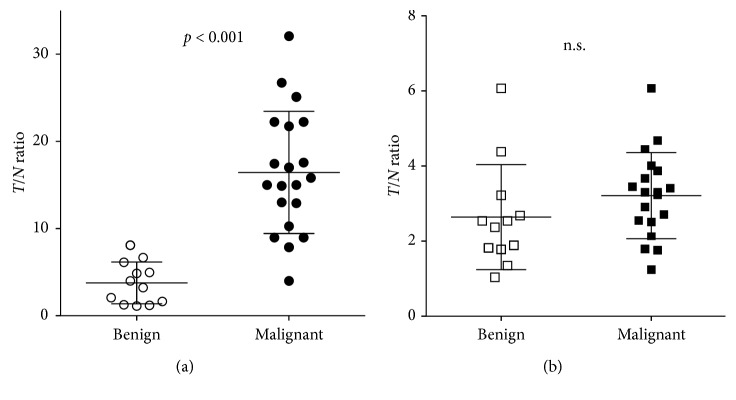
Comparison between benign and malignant groups by *T*/*N* ratio in MeAIB-PET (a) and MET-PET (b).

For the analysis using the *T*/*N* ratio, there was a significant difference between the benign and malignant groups with MeAIB-PET with *p* < 0.001, while no significant difference was observed with MET-PET (Figure 2).

**Figure 3:**
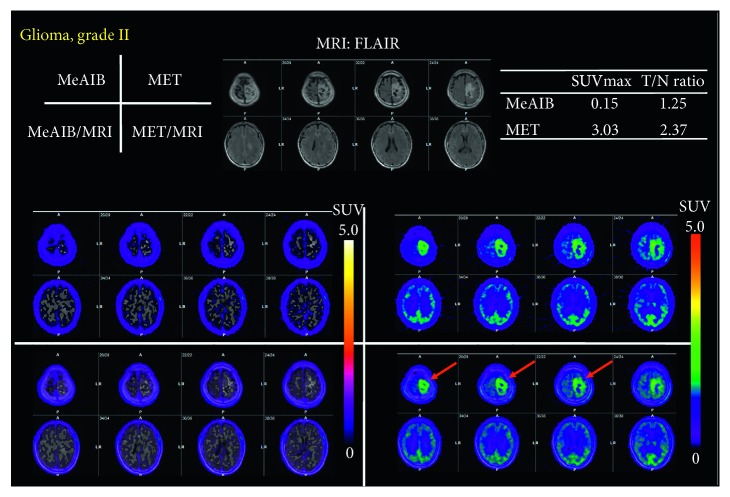
A case of a forty-year-old male who had a diffusely irregular-shaped mass in the left frontal lobe, which was diagnosed as astrocytoma, grade II after surgery (benign group) (case #4). High uptake of MET was in the tumor, while no significant uptake of MeAIB was noted.

**Figure 4:**
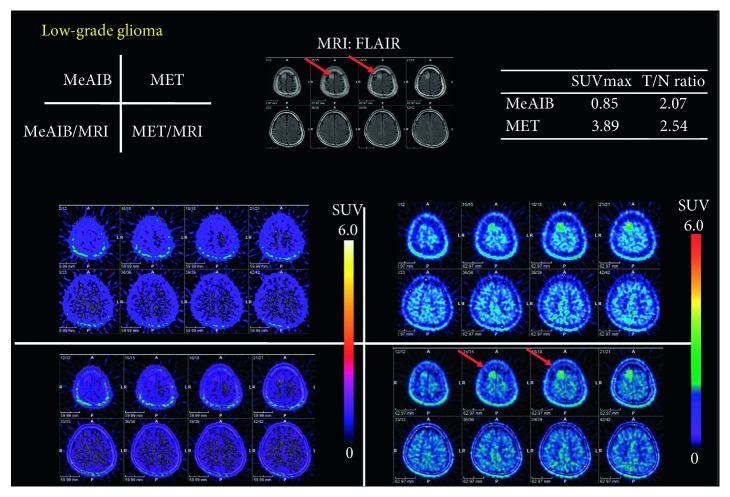
A case of a thirty-three-year-old male having newly diagnosed low-grade glioma in the right frontal lobe by stereotactic biopsy (benign group) (case #6). High uptake of MET was in the tumor, while no significant uptake of MeAIB was noted.

**Figure 5:**
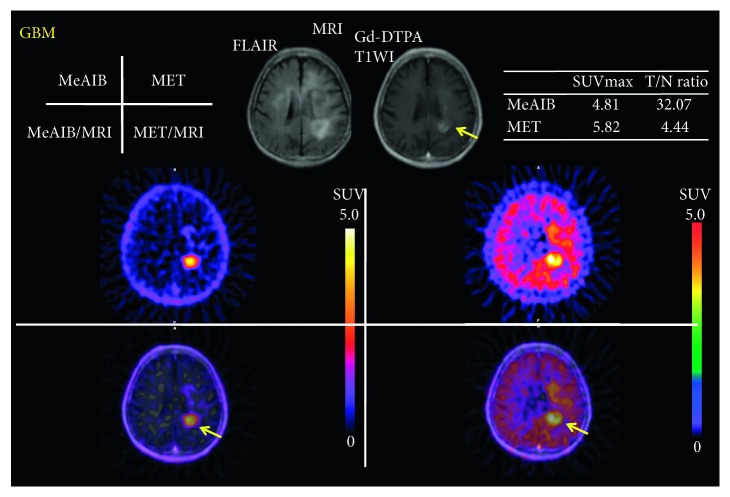
A case of a seventy-one-year-male who had newly diagnosed GBM in the left corpus callosum by stereotactic biopsy (malignant group) (case #13). A clear margined tumor was depicted as a high uptake of MeAIB lesion. MET-PET also demonstrated the lesion with the physiological uptake. Higher *T*/*N* ratio was noted in MeAIB-PET image.

Figures 3 and 4 show typical cases in the benign group, which were diagnosed as astrocytoma grade II and low-grade glioma after surgery or stereotactic biopsy. High uptake of MET was in the tumor, while no significant uptake of MeAIB was noted in both cases. In addition, other typical cases in the malignant group are shown in Figures 5 and 8, which were diagnosed as GBM and recurrent GBM; a clear margined tumor was depicted as a high uptake of MeAIB lesion. MET-PET also demonstrated the lesion with the physiological uptake. Higher *T*/*N* ratio was noted in MeAIB-PET image, respectively.

### 3.3. Diagnostic Accuracies of MeAIB- and MET-PET

**Figure 6:**
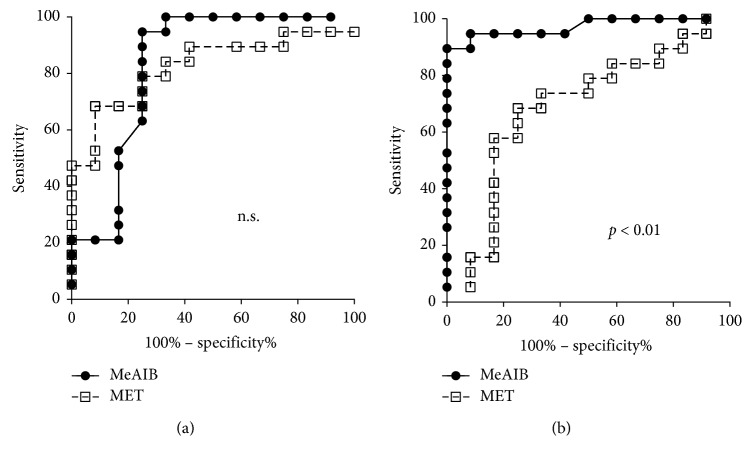
Receiver-operating characteristic curve (ROC) analyses for the diagnostic accuracy of MeAIB-PET and MET-PET using semiquantitative analysis. (a) For ROC analysis using SUVmax, the area under the curve (AUC) value for MeAIB PET was 0.83 with standard error 0.090, 95% CI 0.65–1.00, and *p* < 0.005. The AUC for MET-PET was 0.82 with standard error 0.076, 95% CI 0.67–0.97, and *p* < 0.005. There was no significance of diagnosis accuracy between them. (b) For ROC analysis using *T*/*N* ratio, the AUC value for MeAIB PET was 0.97 with standard error 0.027, 95% CI 0.92–1.02, and *p* < 0.0001. The AUC for MET-PET was 0.69 with standard error 0.10, 95% CI 0.48–0.89, and *p* < 0.1. These analyses indicated better diagnosis accuracy of MeAIB-PET for brain tumors than MET-PET (*p* < 0.01).

As for the differential diagnosis of brain tumors between the benign and malignant groups, receiver operating characteristic curve (ROC) analyses for the diagnostic accuracy of MeAIB-PET and MET-PET using a semiquantitative analysis were assessed (Figure 6). For ROC analysis using SUVmax, the area under curve (AUC) value for MeAIB-PET was 0.83 with standard error 0.090, 95% CI 0.65–1.00, and *p* < 0.005. The AUC for MET-PET was 0.82 with standard error 0.076, 95% CI 0.67–0.97, and *p* < 0.005. There was no significant difference in diagnosis accuracy between them (Figure 6(a)). For ROC analysis using the *T*/*N* ratio, the AUC value for MeAIB-PET was 0.97 with standard error 0.027, 95% CI 0.92–1.02, and *p* < 0.0001. The AUC for MET-PET was 0.69 with standard error 0.10, 95% CI 0.48–0.89, and *p* < 0.1. These analyses indicated a better diagnosis accuracy for MeAIB-PET for brain tumors than MET-PET (*p* < 0.01) (Figure 6(b)).

When the cutoff value was set as SUVmax = 2.0 for MeAIB-PET, the sensitivity, specificity, and accuracy were 73.7%, 91.7%, and 80.6%, respectively, while if the cutoff value was set as SUVmax = 3.5 for MET-PET, the sensitivity, specificity, and accuracy were 73.7%, 75.0%, and 74.2%, respectively. When the cutoff value was set as *T*/*N* ratio = 7.0 for MeAIB-PET, the sensitivity, specificity, and accuracy were 94.7%, 91.7%, and 93.5%, respectively, while if the cutoff value was set as *T*/*N* ratio = 3.0 for MET-PET, the sensitivity, specificity, and accuracy were 57.9%, 75.0%, and 64.5%, respectively.

### 3.4. Relationship of SUVmax in the Lesion with MeAIB and MET

**Figure 7:**
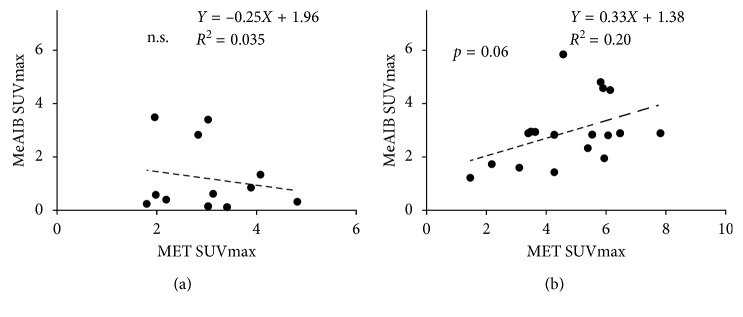
Relationship between SUVmax of MeAIB and that of MET of each lesion in both PET study using logistic regression. In the benign group, SUVmax of MeAIB showed nonsignificant linear relationship with that of MET (a). On the contrary, in the malignant group, SUVmax of MeAIB showed a weak positive correlation with that of MET (*p*=0.06, *R*^2^=0.20) (b).

Relationships between SUVmax of MeAIB and that of MET of each lesion in both PET studies using logistic regression are shown in Figure 7. In the benign group, the SUVmax of MeAIB showed a nonsignificant linear relationship with that of MET (Figure 7(a)). On the contrary, in the malignant group, the SUVmax of MeAIB showed a weak positive correlation with that of MET (*p*=0.06, *R*^2^=0.20) (Figure 7(b)).

## 4. Discussion

System A amino acid transport is Na^+^- and energy-dependent, highly concentrative, and a putative regulator of cell growth. Malignant transformation is associated with enhanced system A activity [22]. System A is specifically capable of transporting *N*-methylated amino acids [23]. The amino acid analog MeAIB was developed as an ideal tracer for in vivo transport measurements, as the compound is nonmetabolizable and concentrated in cells only via system A transport [15, 20]. There are several reports regarding clinical MeAIB-PET in patients with lymphoma [14], head and neck cancer [16], in addition to our previous study on pulmonary and mediastinal mass lesions [18], and prostate cancer [19]. However, there have been no reports evaluating the usefulness of MeAIB-PET for the diagnosis of brain tumors.

**Figure 8:**
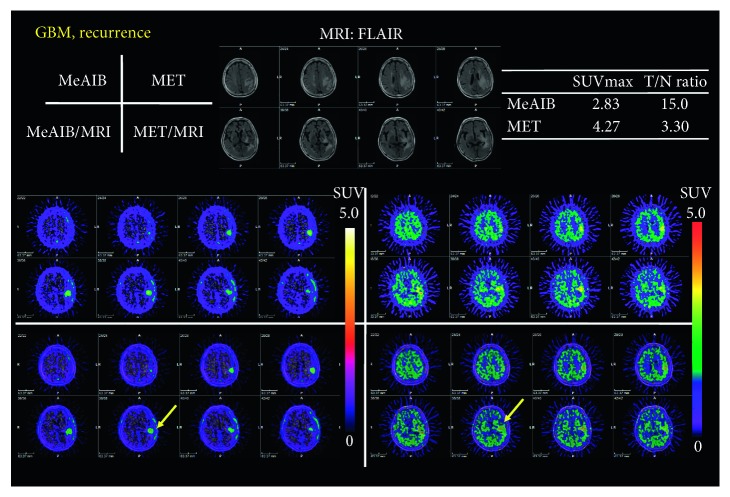
A case of a sixty-eight-year-old female who had received chemoradiotherapy before for GBM in the left temporal lobe (malignant group) (case #18). A clear margined tumor was depicted as a high uptake of MeAIB lesion. MET-PET also demonstrated the lesion; however, the margin of the lesion was unclear due to the physiological uptake of MET in the brain. Recurrent GBM was confirmed after stereotactic biopsy.

Our principal finding is that the diagnostic accuracy of MeAIB using *T*/*N* ratio was better than those of MET-PET when differentiating benign and malignant brain lesions. The *T*/*N* ratio with MeAIB-PET was higher than that with MET because of the faint uptake of MeAIB by normal brains (Figures 3–5 and 8). The reason for this is that MeAIB has difficulty permeating the blood-brain barrier (BBB) [24]. Using the *T*/*N* ratio, MeAIB-PET displayed higher diagnostic accuracy in distinguishing between the benign and malignant groups (Figure 6), which resulted in showing relatively low false negative findings than MET-PET. Therefore, MeAIB-PET may be useful for the diagnosis of malignant brain tumors with broken BBB and high expressions of system A transport. This high *T*/*N* ratio may result in the clear contrast between surrounding brain tissues and the marginal edge of malignant brain tumor. Moreover, MeAIB-PET may contribute to more accurate depictions of the tumor margin when stereotactic surgery/biopsy or stereotactic radiotherapy is considered for the treatment of malignant brain tumors. The uptake of MeAIB in brain tumors was lower than that of MET. This may represent a difference in expression between system A and L amino acid transport in tumors. Considering the results of the relationships between SUVmax of MeAIB and that of MET for each tumor in the malignant group, SUVmax of MeAIB showed a weak linear relationship, and not significant, with that of MET.

MET is mainly transported by system L amino acid transporters. MET-PET has been widely used for brain tumor imaging [25–28]. However, for tumor grading using MET-PET, Hatakeyama et al. reported that the differences of MET SUVmax and *T*/*N* ratios between grades II and III gliomas were not statistically significant and that low-grade gliomas with oligodendroglial components had relatively high MET uptake [25]. Sasaki et al. noted that MET was highly useful both for detecting astrocytoma and for differentiating between benign and malignant astrocytomas. However, it was not sufficiently useful to evaluate the histological grade of the astrocytomas [27]. In the present study, although MET-PET showed the possibility of distinguishing between low-grade and high-grade astrocytoma using SUVmax, the *T*/*N* ratio was not useful for tumor grading (Figure 2), as suggested in previous reports. Physiological uptake of MET by the brain via system L amino acid transporters is considered to be one of the reasons for this result because MET is known to be utilized for the physiological metabolism of normal brain tissues as a substrate for protein synthesis, neurotransmitters, and energy production [2, 10, 11, 29]. In this study, indeed, MET-PET had a tendency to show false positive findings more than MeAIB-PET. The physiological uptake of MET by the normal brain tissue is considered as one of the reasons. In considering other amino acid PET imaging, Inoue et al. investigated an amino acid PET imaging using L-3-[F-18]-fluoro-alpha-methyl tyrosine (FAMT) and reported the mean value of SUV of the brain tumor as 2.83 ± 1.57 in FAMT-PET [30]. This is similar to the result of MET-PET because FAMT is transported into cancer cells via system L amino acid transporter [31].

In terms of the study limitations, there were a relatively small number in the study population that participated in this study. Most of them are astrocytoma grade II or less and GBM. And there were ten recurrent GBM in addition to newly diagnosed GBM included in this study. Recurrent tumors analyzed in this study were lesions 6 months or more after successful treatment, so there might be little effect of the treatment for analysis of this PET imaging study. However, detailed examination with large number of subjects was considered to be needed in terms of influences after surgery or chemotherapy on PET image. Moreover, further study is also needed, including that of brain tumors other than astrocytic tumors such as metastatic brain tumors or CNS lymphoma.

## 5. Conclusions

We investigated system A amino acid transport PET imaging, MeAIB-PET, in patients with astrocytoma and GBM and compared the diagnostic results to those obtained by MET-PET. MeAIB-PET could provide better assessments for detecting malignant-type brain tumors. In a differential diagnosis between low-grade and high-grade astrocytoma, MeAIB-PET is a useful diagnostic imaging tool, especially in evaluations using the *T*/*N* ratio.

## Figures and Tables

**Table 1:** Patient characteristics.

Total (*n*=31)	
Age (years)	
Mean ± SD	44.2 ± 18.5
Median	44
Range	5–71
Male : female	16 : 15

Group A (benign) (*n*=12)	
Age (years)	
Mean ± SD	32.2 ± 10.0
Median	33.5
Range	5–46
Male : female	7 : 5
Diagnosis	
Astrocytoma grade II or less/low-grade glioma	11
Brain stem glioma	1

Group B (malignant) (*n*=19)	
Age (years)	
Mean ± SD	56.7 ± 16.8
Median	60
Range	14–71
Male : female	9 : 10
Diagnosis	
Glioblastoma multiforme	7
Glioblastoma multiforme, recurrence	10
Anaplastic astrocytoma, grade 3	2

**Table 2:** SUVmax and *T*/*N* ratios of MeAIB- and MET-PET study in patients with brain tumors.

*Group A (benign group)*					
Diagnosis		MeAIB		MET	
		SUVmax	*T*/*N* ratio	SUVmax	*T*/*N* ratio
1	Low-grade glioma	0.58	3.22	1.98	1.78
2	Astrocytoma grade II	2.83	8.09	2.83	1.35
3	Low-grade glioma	0.24	1.64	1.80	1.04
4	Glioma grade II	0.15	1.25	3.03	2.37
5	Low-grade glioma	0.62	1.11	3.13	1.82
6	Low-grade glioma	0.85	2.07	3.89	2.54
7	Brain stem glioma	3.40	4.86	3.03	1.89
8	Glioma grade II, rec.	3.49	6.13	1.96	2.68
9	Low-grade glioma	0.32	4.00	4.82	4.38
10	Low-grade glioma	0.4	6.67	2.19	3.22
11	Low-grade glioma	1.34	4.96	4.08	6.07
12	Low-grade glioma	0.12	1.20	3.41	2.54
Ave.		1.20	3.77	3.01	2.64

*Group B (malignant group)*					
13	GBM	4.81	32.07	5.82	4.44
14	GBM, rec.	2.95	14.89	3.49	3.45
15	GBM, rec.	2.94	26.72	3.63	3.67
16	GBM, rec.	2.33	8.96	5.39	2.51
17	GBM, rec.	2.84	12.91	5.54	2.55
18	GBM, rec.	2.83	15.00	4.27	3.30
19	GBM	1.60	4.00	3.10	1.24
20	GBM	2.89	22.23	6.47	3.87
21	GBM, rec.	4.51	25.10	6.14	3.23
22	GBM, rec.	2.89	17.00	3.40	2.91
23	GBM	2.81	17.56	6.07	6.07
24	GBM	5.85	15.81	4.57	3.41
25	GBM, rec.	1.22	17.43	1.46	1.76
26	GBM, rec.	4.58	21.73	5.90	2.71
27	GBM, rec.	2.83	15.00	4.27	3.30
28	GBM	2.89	22.23	7.82	4.68
29	Anaplastic astrocytoma	1.95	10.26	5.94	4.01
30	Anaplastic astrocytoma	1.73	7.86	2.18	1.79
31	GBM	1.43	13.00	4.27	2.12
Ave.		2.94	16.83	4.72	3.21

## Additional Points

*Highlights*.  The uptake of MeAIB in brain tumors was lower but showed higher image contrast than those of MET. Using the *T*/*N* ratio, MeAIB-PET showed higher diagnostic accuracy compared to MET-PET. MeAIB-PET contributes to more accurate depictions of the tumor margin. MeAIB-PET provides better assessments for detecting malignant brain tumors.
